# The endoplasmic reticulum protein HSPA5/BiP is essential for decidual transformation of human endometrial stromal cells

**DOI:** 10.1038/s41598-024-76241-z

**Published:** 2024-10-29

**Authors:** Laura Fernández, Chow-Seng Kong, Majd Alkhoury, Maria Tryfonos, Paul J. Brighton, Thomas M. Rawlings, Joanne Muter, Maria Soledad Gori, Claudia Pérez Leirós, Emma S. Lucas, Jan J. Brosens, Rosanna Ramhorst

**Affiliations:** 1 CONICET, Universidad de Buenos Aires, Instituto de Química Biológica de la Facultad de Ciencias Exactas y Naturales IQUIBICEN, Buenos Aires, Argentina; 2 Warwick Medical School, Division of Biomedical Sciences, University of Warwick, Coventry, UK; 3 Tommy’s National Centre for Miscarriage Research, University Hospitals Coventry and Warwickshire NHS Trust, Coventry, CV2 2DX UK; 4 School of Sciences, University of Buenos Aires, IQUIBICEN-CONICET, Int. Guiraldes 2160, Ciudad Universitaria, Pabellón 2 Piso 4, C1428EHA Buenos Aires, Argentina; 5 Faculty of Health, University of Sheffield, Sheffield, UK

**Keywords:** Molecular medicine, Translational research, Endocrine system

## Abstract

Decidualization denotes the process of inflammatory reprogramming of endometrial stromal cells (EnSC) into specialized decidual cells (DC). During this process, EnSC are subjected to endoplasmic reticulum (ER) stress as well as acute cellular senescence. Both processes contribute to the proinflammatory mid-luteal implantation window and their dysregulation has been implicated in reproductive failure. Here, we evaluated the link between ER stress, decidual differentiation and senescence. *In-silico* analysis identified *HSPA5* gene, codifying the ER chaperone BiP, as a potentially critical regulator of cell fate divergence of decidualizing EnSC into anti-inflammatory DC and pro-inflammatory senescent decidual cells (snDC). Knockdown of *HSPA5* in primary EnSC resulted both in decreased expression of DC marker genes and attenuated induction of senescence associated β-galactosidase activity, a marker of snDC. Stalling of the decidual reaction upon *HSPA5* knockdown was apparent at 8 days of differentiation and was preceded by the upregulation of ER stress associated proteins IRE1α and PERK. Further, *HSPA5* knockdown impaired colony-forming unit activity of primary EnSC, indicative of loss of cellular plasticity. Together, our results point to a key role for *HSPA5*/BiP in decidual transformation of EnSCs and highlight the importance of constraining ER stress levels during this process.

**Supplementary Information:**

The online version contains supplementary material available at 10.1038/s41598-024-76241-z.

## Introduction

The opening mid-luteal window of implantation coincides with abrupt changes in endometrial gene expression, indicative of an acute tissue stress response^1^. Histologically, this stress response, termed decidual reaction, is characterized by the onset of glandular secretion, increased vascular perfusion and local edema, proliferative expansion of uterine natural killer cells (uNK), and decidualization (differentiation) of endometrial stromal cells (EnSC)^2^. Decidualizing EnSC progressively lose their fibroblastic appearance and acquire an epithelioid morphology with abundant cytoplasm and prominent endoplasmic reticulum (ER)^3–5^.

Alongside the morphological alterations, decidualization involves extensive reprogramming of multiple signaling pathways and cellular functions, including acquisition of a prominent secretory phenotype^2,6^. Most of the secreted and membrane proteins are translated in ribosomes associated to the ER where proteins undergo folding and assembly^7^. Then, the requirement for increased protein secretion upon decidual transformation imposes ER stress and triggers the unfolded protein response (UPR) in EnSC^8–10^. Heat shock protein family A (Hsp70) member 5, also known as BiP, is a chaperone protein that plays a critical role in maintaining ER homeostasis^11^. Under physiological conditions, BiP binds and inactivates the ER stress sensors PERK, IRE1α and ATF6α. When the ER folding machinery becomes overwhelmed, so-called ER-stress, unfolded proteins accumulate in the luminal side of the ER. This leads to BiP release, activating the sensor proteins and triggering the UPR^7^. The UPR pathways promote degradation of misfolded proteins, attenuation of translation and upregulation of chaperone proteins to restore ER homeostasis^12,13^. However, if ER stress levels remain uncontrolled, the UPR elicits a proinflammatory response that may cause cell death^14^. The relevance of ER stress and UPR during the peri-implantation period has been demonstrated in previous studies. Decidualization of human EnSC in vitro is accompanied by upregulation of ER stress sensor proteins ATF6, PERK and IRE1α, as well as UPR markers^8,10^. Moreover, IRE1α knockout mice die on embryonic day 12.5 due to an impaired placental labyrinth layer^15^. Additionally, inhibition of ATF6α pathway has been shown to preclude embryo implantation in animal models^16^.

Single-cell RNA sequencing (scRNA-seq) analysis demonstrated that decidual differentiation is a time-dependent process^17^. While decidual transformation of EnSC in vivo coincides with the beginning of the implantation window, the emergence of a phenotype characteristic of decidual cells (DC) approximately four days later heralds the closure of the window^2^. Similarly, decidualization of primary cultures in vitro involves highly coordinated time-sensitive gene expression changes, reflecting inflammatory reprogramming of EnSC. By the end of this process, however, two distinct subpopulations emerge: progesterone-dependent DC and progesterone-resistant senescent decidual cells (snDC)^18^. Experimental evidence demonstrated that snDC arise from EnSC subjected to excessive replication stress in response to rapid estrogen-dependent proliferation^19,20^. Senescence is defined by a state of permanent cell-cycle arrest, heightened metabolism and abundant secretion of extracellular matrix proteases, proinflammatory cytokines, chemokines, growth factors and reactive oxygen species^21,22^. These molecules are referred to as senescenceassociated secretory phenotype (SASP). Accumulation of chronic senescent cells causes sterile inflammation and loss of tissue function and integrity, a hallmark of ageing and age-related pathologies. By contrast, acute or transient senescence is widely implicated in developmental processes and tissue remodeling^23^. In the endometrium, acute cellular senescence of a discrete number of EnSC has been linked to spontaneous decidualization at the start of the implantation window^2^. Under continuous progesterone signaling, DC in turn recruit and activate uterine natural killer cells in an IL15-dependent manner, which prevents accumulation of chronic snDCs through targeted killing^20^. Thus, a balance between decidual subsets has been proposed to be key for optimal implantation as both lack of acute senescent cells and SASP-associated inflammatory signals and accumulation of snDC have been implicated in reproductive failure^18,20,24,25^.

The mechanisms that control cell fate divergence of EnSC to anti-inflammatory DC and proinflammatory snDC are not completely understood^24^. Given that the SASP production is a canonical feature of senescent cells, we focused on the role of ER stress and UPR in regulating decidual senescence in primary EnSC cultures. We demonstrate that knockdown of *HSPA5*/BiP in primary culture is sufficient to stall the decidual reaction by disrupting the reprogramming of EnSC into diverging DC and snDC subsets.

## Results

### Expression of ER stress pathway genes in EnSC decidualizedin vitro

**Fig. 1:**
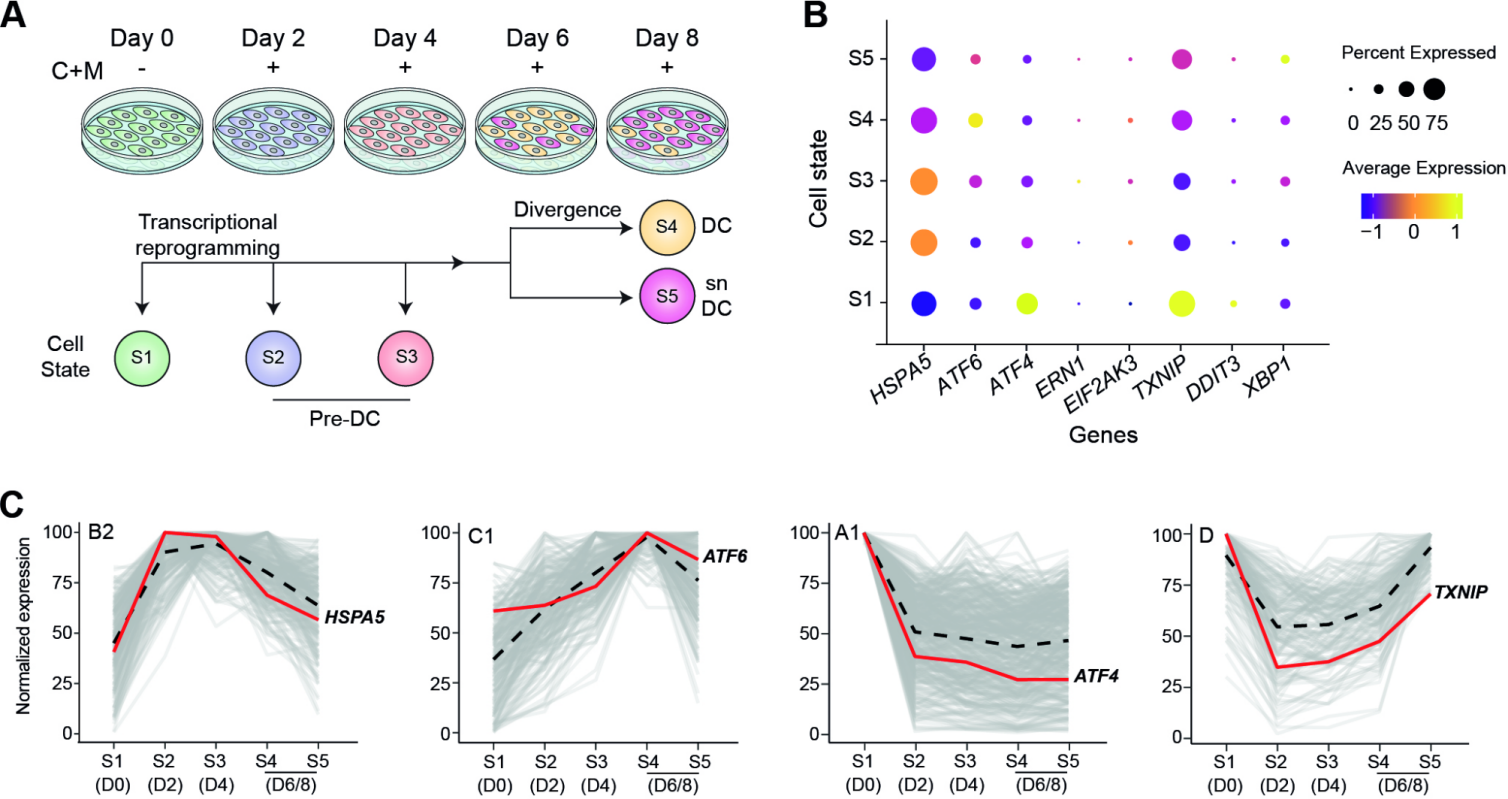
Single cell expression of ER stress/UPR-associated genes during the decidualization process. (**A**) Schematic representation of in vitro decidualization of EnSCs and the progress of transcriptional states along time. (**B**) Expression of *HSPA5*, *ATF6*, *ATF4*, *ERN1*, *EIF2AK3*, *TXNIP*, *DDIT3* and *XBP1* was evaluated in silico on decidualizing EnSCs using scRNA sequencing data from Lucas et al.^18^. Color intensity indicates average gene expression, while circle size shows the percentage of cells expressing each gene. (**C**) Co-regulated decidual networks for *HSPA5*, *ATF6*, *ATF4* and *TXNIP*. B2, C1, A1 and D indicates the gene clusters from Lucas et al.^18^.

We set out to evaluate the interplay between ER stress and cellular senescence, two processes implicated in decidualization and reproductive disorders^8,18^. We first mined published single-cell RNAsequencing (scRNAseq) data that span the decidual pathway in vitro (GEO Datasets accession number: GSE127918)^18^. Briefly, primary EnSC were decidualized in vitro with 8-bromoadenosine 3′,5′-cyclic adenosine monophosphate and medroxyprogesterone (C + M) in a timecourse experiment lasting 8 days. The cultures were harvested every 48 h for scRNA-seq analysis. Figure 1A provides a schematic overview of the five distinct transcriptional cell states identified across the timecourse. Cell state 1 (S1) marks undifferentiated EnSC at the start of the decidual timecourse (day 0). In response to a deciduogenic stimulus, EnSC undergo time-sensitive transcriptional reprogramming, a process that takes approximately four days^18,19,26^. The transcriptomic profiles of these differentiating cells, termed pre-decidual cells (pre-DC), differ markedly between day 2 (S2) and day 4 (S3) of the decidual timecourse^18^. The temporal changes in gene expression are lost after day 4, with the emergence of DC (S4) and a discrete population of snDC (S5) on day 6. In the absence of snDC clearence by immune cells, SASP-mediated paracrine senescence accounts for the expansion of snDC at the expense of DC by day 8 of the decidual timecourse (Fig. 1A)^18^.

We analyzed the expression of the ER stress/UPR-associated genes, including *HSPA5* (codifying for ER chaperone BiP) *ERN1*,* EIF2AK3* and *ATF6* (encoding the ER stress sensors IRE1α, PERK and ATF6, respectively), *ATF4*, *XBP1* and *DDIT3* (mediators of the adaptative UPR), and *TXNIP* (thioredoxin interacting protein, a regulator of cellular metabolism and the inflammatory UPR pathway)^27^. Figure 1B demonstrates that four genes, *HSPA5*, *ATF6*, *ATF4* and *TXNIP*, are abundantly expressed in a temporal manner across the decidual timecourse. As the temporal trajectory of expression differed for each of the four genes, we mapped the profiles onto our previously reported time-dependent coregulated gene clusters^18^. As shown in Fig. 1C, each gene belongs to a different group of coregulated transcripts: *HSPA5* belongs to a network of biphasic genes that peak prior to the divergence of pre-DC into DC and snDC; *ATF6* belongs to genes gradually induced upon decidualization with peak expression in DC; *ATF4* is part of a group of genes firmly repressed across the decidual pathway; and *TXNIP* belongs to a group of genes that are rapidly downregulated upon decidualization but then reexpressed, predominantly in progesterone-resistant snDC.

### Expression of ER stress pathway genes in EnSC across the luteal phase

**Fig. 2:**
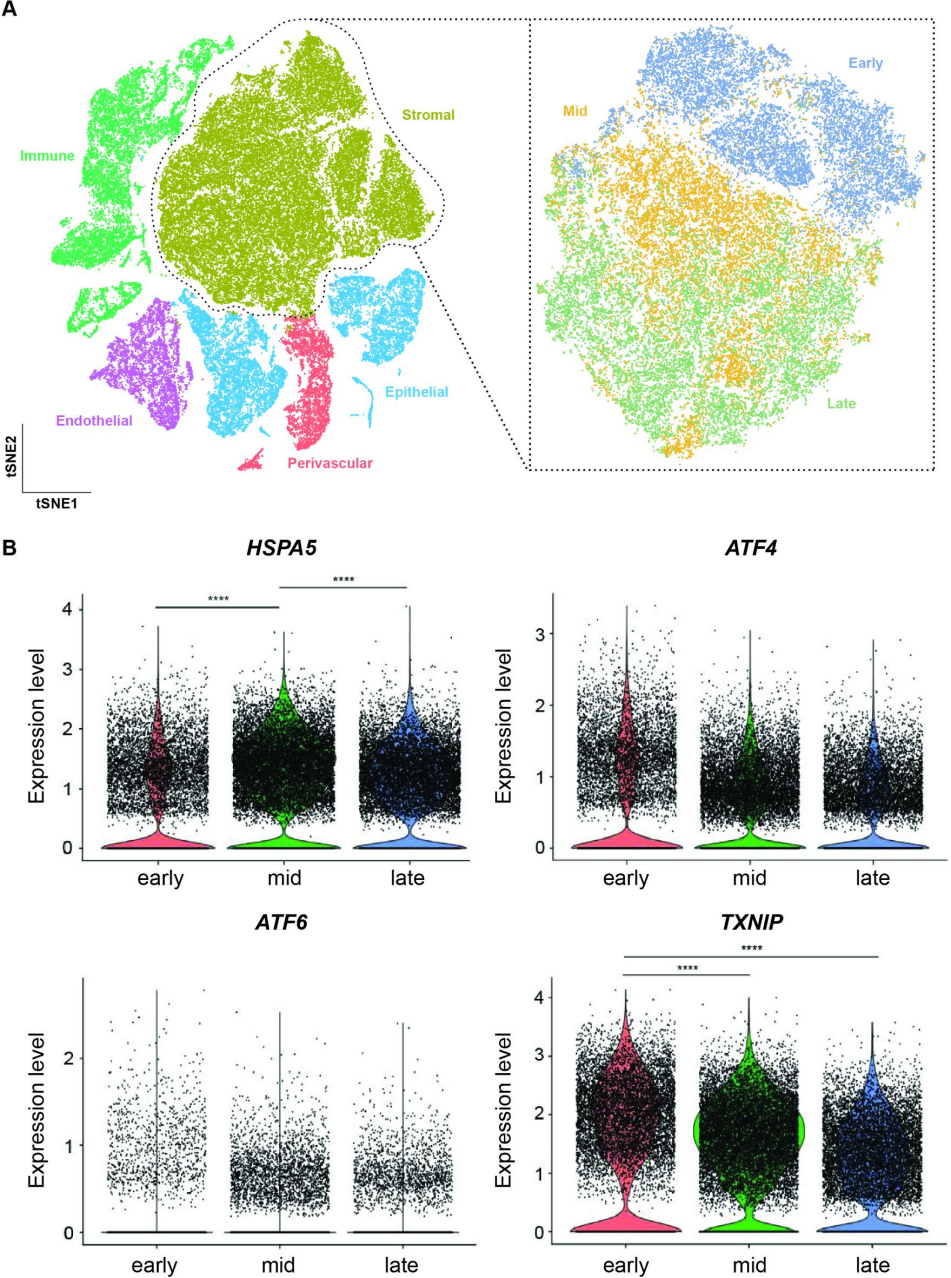
Single cell expression of ER stress/UPR-associated genes on endometrial stromal cells across the luteal phase. (**A**) *t*-SNE plot of different cell subpopulations from 12 endometrial samples taken across the luteal phase. (**B**) Expression of *HSPA5*, *ATF6*, *ATF4* and *TXNIP* was in silico evaluated on the stromal subset on cells from early, mid or late secretory phase. Statistical significance of the differences was evaluated by Wilcoxon rank sum test, ****p < 0.0001.

Next, we examined the expression of the same set of four genes (*HSPA5*, *ATF4*, *ATF6* and *TXNIP*) in EnSC across the luteal phase in vivo. For this purpose, we mined scRNA-seq data of endometrial biopsies obtained at different timepoints after the pre-ovulatory luteinizing hormone (LH) surge, including 4 early-secretory phase (LH + 5–6 days) samples, 5 samples obtained in the mid-secretory window of implantation (LH + 7–9 days), and 3 late-secetory phase (LH + 10–11 days) biopsies (GEO Profiles accession number: GSE247962). Following quality control, Shared Nearest Neighbour (SNN) and *t*Distributed Stochastic Neighbour Embedding (*t*-SNE) analysis of 64,644 cells revealed 6 major clusters (Fig. 2A). Based on the expression of canonical marker genes, these cell clusters corresponded to endothelial cells (*n* = 4701), epithelial cells (*n* = 10,280), ciliated epithelial cells (*n* = 572), EnSC (*n* = 33,416), perivascular cells (*n* = 3950) and immune cells (*n* = 11,725). For the purpose of this study, expression analysis was restricted to EnSC. As shown in Fig. 2B, *HSPA5* transcript levels peaked in the midluteal phase of the cycle, in keeping with the expression profile observed across the decidual pathway in vitro. Further, progression across the luteal phase was associated with downregulation of *TXNIP* expression, again in keeping with the in vitro timecourse profile. In addition, *ATF4* and *ATF6* expression appeared to decline upon opening of the midluteal implantation window, although the changes were not statistically significant (FDR corrected *p* > 0.05).

### Loss of HSPA5/BiP elicits an unfolded protein response in decidualizing EnSC

**Fig. 3:**
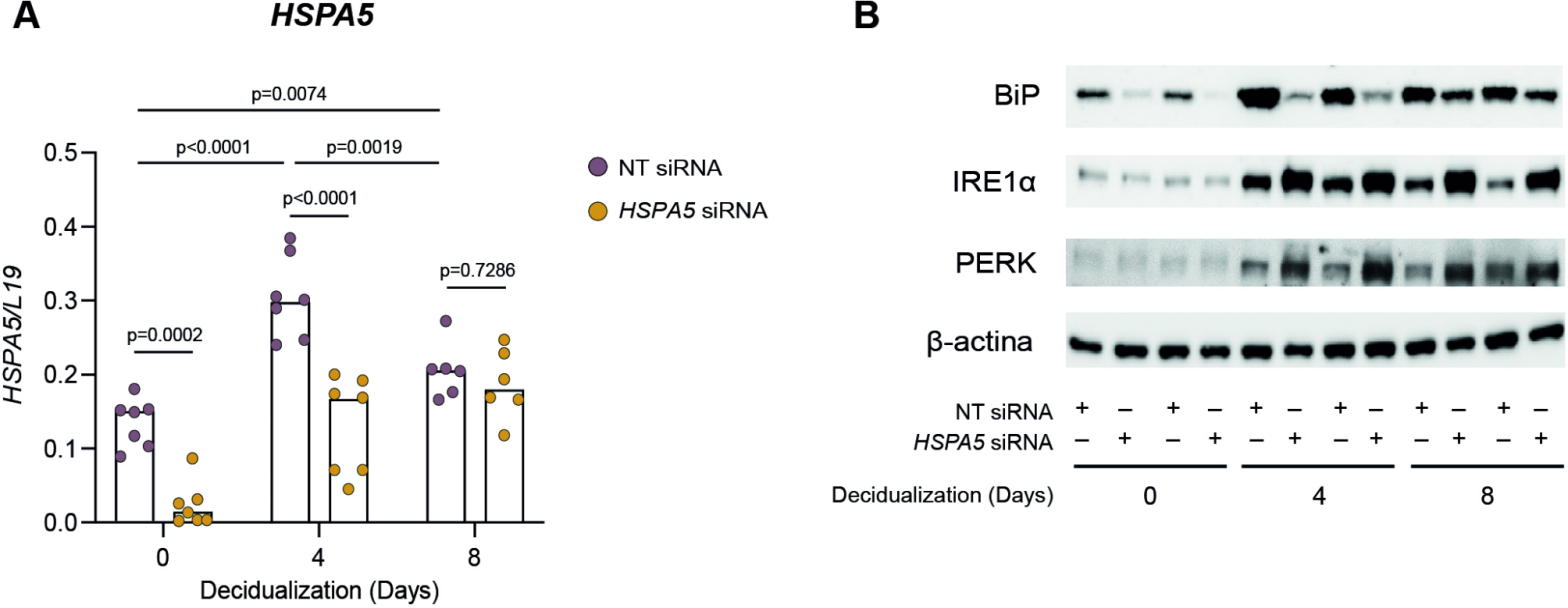
*HSPA5* knockdown induces ER stress in decidualizing EnSCs. EnSCs were purified from endometrial samples and *HSPA5* was knocked down by siRNA transfection. Non-targeting siRNA (NT) was used as control. (**A**) *HSPA5* knockdown was confirmed in undifferentiated and decidualized cells at mRNA level by RTqPCR. Individual data points from 7 biological replicates are shown, with bar graphs indicating the median. Statistical differences were evaluated by Mixed-effect analysis. (**B**) The impact of *HSPA5* knockdown on protein levels of BiP, as well as the ER stress markers IRE1α and PERK, was examined by western blotting in 2 biological replicates. β-Actin was used as a loading control. The grouping of gels/blots showed correspond to the full-length.

Our expression analyses demonstrated that *HSPA5* expression is tightly regulated across the decidual pathway, characterized by maximal expression during the midluteal implantation window in vivo and upon transition of pre-DC into DC and snDC subsets in vitro. Considering that HSPA5/BiP binding to ER stress sensor proteins prevents the triggering of the UPR response^7,11^, we evaluated the effect of *HSPA5* silencing on this pathway in decidualizing EnSC. A total of 7 primary cultures established from different endometrial biopsies were transiently transfected with either non-targeting (NT) or *HSPA5* siRNA. There was no evidence that loss of *HSPA5*/BiP impacted on cell viability (Supplementary Fig. 1). After a recovery period of 24 h, the cultures were decidualized for 4 or 8 days. Total RNA and protein were extracted from undifferentiated (day 0) and decidualizing cultures (day 4 and day 8). Consistent with the scRNA-seq data (Fig. 1B), *HSPA5* mRNA expression in control cultures transfected with NT siRNA was induced significantly upon decidualization with levels peaking on day 4 (median 2.02-fold induction, *p* < 0.0001) (Fig. 3A). Transcript levels subsequently declined as the decidualization timecourse progressed to day 8 of differentiation (*p* = 0.0019). Western Blot analysis of two independent cultures confirmed that expression of *HSPA5* at mRNA level corresponds to protein levels across the decidual timecourse (Fig. 3B). Figure 3A also shows siRNA-mediated *HSPA5* knockdown was highly efficient in undifferentiated cells (11.46-fold reduction, *p* = 0.0002). *HSPA5* silencing was partially maintained on day 4 of decidualization (1.79-fold reduction, *p* < 0.0001), but lost by day 8 (*p* = 0.7286). However, Western blot analysis not only confirmed *HSPA5*/BiP knockdown, but also showed that expression at protein level is not fully restored by day 8 of decidualization (Fig. 3B). Further, *HSPA5* knockdown increased the protein levels of IRE1α and PERK during decidualization, suggesting activation of the UPR pathway (Fig. 3B). Interestingly, no discernible changes were observed in either IRE1α or PERK protein levels in undifferentiated cells (day 0), suggesting that loss of *HSPA5*/BiP is more consequential in the context of the decidualization and associated with ER stress. Since an exacerbated ER stress response can induce cell death^28,29^, we confirmed that cell viability was not altered by *HSPA5* siRNA after 8 days of decidualization (Supplementary Fig. 2).

### *HSPA5* knockdown impairs the transition of pre-decidual cells into decidual subsets

**Fig. 4:**
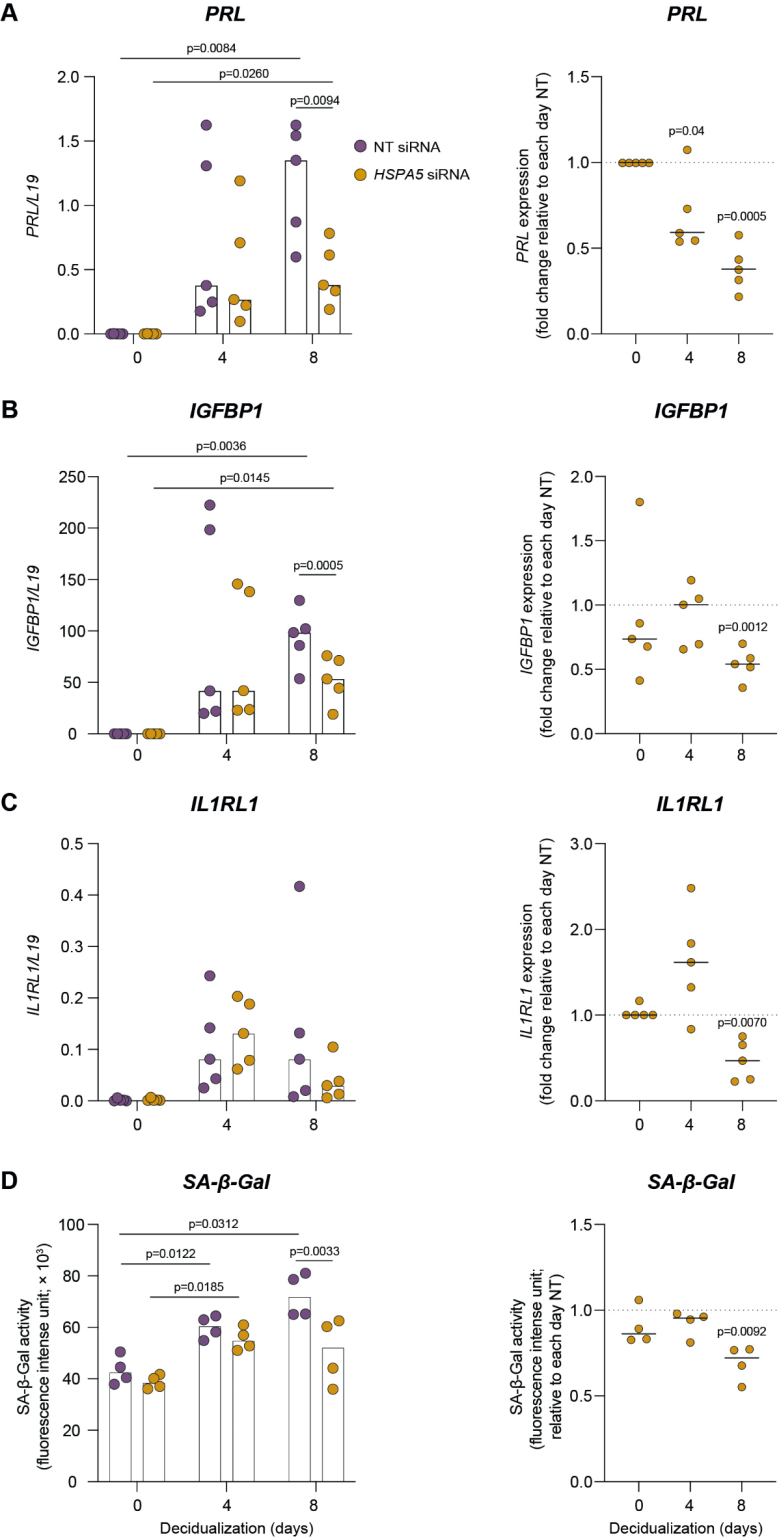
*HSPA5* silencing impedes the emergence of mature and senescent decidual cells. EnSCs were transfected with *HSPA5* or NT siRNA and then decidualized in vitro. Expression of decidualization markers *PRL* (**A**) and *IGFBP1* (**B**) were tested by RTqPCR. (**C**) To evaluate the emergence of mature decidual cells, *IL1RL1* expression was evaluated by RTqPCR. (**D**) To assess the emergence of senescent decidual cells, βgalactosidase activity was tested. The left panels show expression levels of indicated genes normalized to L19, whereas the right panels show fold-change upon transfection of *HSPA5* siRNA compared to control cultures transfected with NT siRNA at the indicated timepoints. Individual data points from 4–5 biological replicates are shown, with bar graphs indicating the median. Statistical significance of the differences was evaluated by Two-way ANOVA (left panels) and One-way ANOVA (right panels).

Next, we examined the effect of *HSPA5*/BiP knockdown on the expression of decidual marker genes. EnSCs were transfected with *HSPA5* or NT siRNA and decidualized with C + M for 4 or 8 days. *HSPA5* knockdown significantly decreased the expression of canonical decidualization markers *PRL* and *IGFBP1* on day 8 of decidualization (*p* = 0.0005 and *p* = 0.0012, respectively; Fig. 4A,B). *IL1RL1*, encoding the transmembrane and soluble IL-33 receptor, represents a DC-specific marker^18,26^. As shown in Fig. 4C, *HSPA5* knockdown significantly decreased the expression of this marker gene at the same time point (*p* = 0.0070). Senescence-associated β-galactosidase activity, a marker of cellular senescence used to evaluate emerging snDC, was also significantly lower upon decidualization of cultures first transfected with *HSPA5* siRNA when compared to the corresponding NT control (*p* = 0.0092; Fig. 4D). Overall, these results suggest that induction of *HSPA5* in pre-decidual cells is essential for progression of the decidual pathway and divergence of cells into DC and snDC subpopulations.

### *HSPA5* knockdown compromises EnSC plasticity

**Fig. 5:**
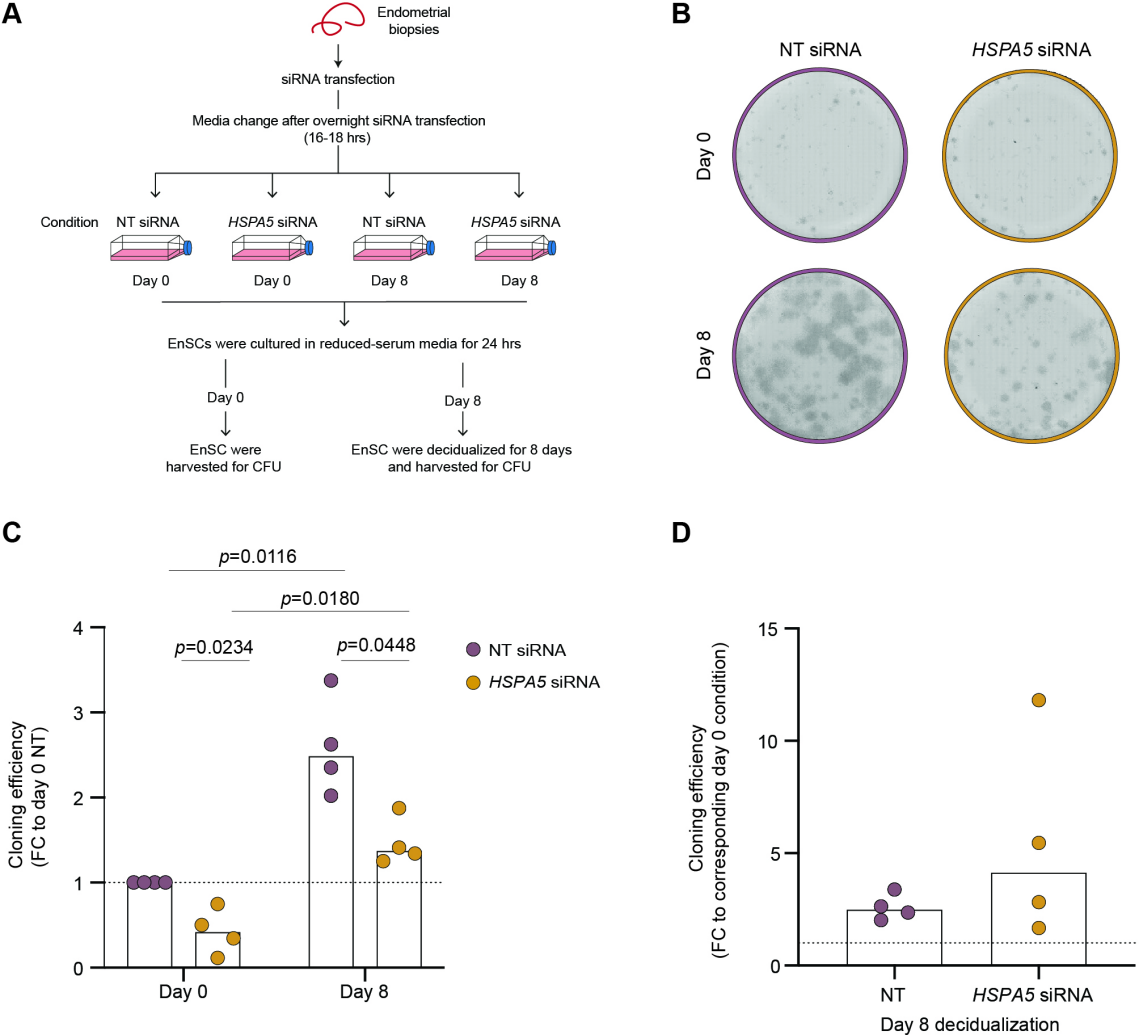
*HSPA5* silencing alters clonogenic activity in undifferentiated EnSCs. EnSCs were transfected with *HSPA5* or NT siRNA and colony forming unit (CFU) activity was evaluated in undifferentiated (C + M Day 0) or decidualized cells (C + M Day 8). (**A**) Schematic representation of the experimental design. (**B**) Representative CFU established from each experimental condition. (**C**) Cloning efficiency in EnSC expressed as fold-change (FC) to the undifferentiated control culture transfected with NT siRNA. (**D**) Cloning efficiency also shown for each *HSPA5* siRNA culture relative to the NT counterpart for the same timepoint. Statistical significance of the differences was evaluated by Two-way ANOVA (**C**) and One-way ANOVA (**D**).

Acute sterile inflammation is not confined to the cycling endometrium but implicated in a myriad of physiological processes, ranging from development to wound healing and tissue repair. In all these processes, acute - but not chronic - stress drives cellular plasticity, defined by dedifferentiation of committed cells into clonogenic mesenchymal stemlike progenitor cells (MSC). Likewise, decidualization has been shown to enhance clonogenicity of primary EnSC, as measured by colonyforming unit (CFU) activity^20^. We subjected undifferentiated and decidualized primary EnSC cultures, transfected with either *HSPA5* or NT siRNA, to CFU assays. (Fig. 5A,B). Unexpectedly, *HSPA5* knockdown compromised CFU activity in undifferentiated EnSC and resulted in lower activity levels upon decidualization (Fig. 5C). However, despite the apparent loss of cellular plasticity in EnSC transfected with *HSPA5* siRNA, the relative increase in CFU activity upon decidualization was unaffected (Fig. 5D). Thus, loss of HSPA5/BiP in undifferentiated EnSC may lead to lack of clonogenic MSC in the decidualizing endometrium during the implantation window.

## Discussion

Decidualization is a tissue specific differentiation of endometrial stromal cells into epithelial-like secretory cells. From an immunological perspective, it is a multistep process that starts with an acute pro-inflammatory response that allows endometrial receptivity and embryo invasion^30^. The duration of this period is tightly controlled, lasting between 2 and 4 days^10,31^. The subsequent switch to an anti-inflammatory response at the closure of the implantation window is mediated by the emergence of mature decidual cells and the recruitment and activation of uterine NK cells to clear senescent cells which constrain inflammation. This step sustains the transition of the decidua to a semi-permanent tissue critical to support the ongoing pregnancy and the maternal tolerance to the fetus^32,33^. However, in the absence of an implanting embryo, the levels of progesterone falling down and the propagation of senescence through the endometrium are associated with influx of neutrophils and macrophages, resulting in tissue breakdown and menstruation^34,35^.

The inflammatory response characteristic of the implantation window has been linked to the ER stress, UPR response and senescence that EnSCs undergo during decidualization^8,10,18,20^. Here, by means of in silico and in vitro designs, we point to the role of ER stress and UPR in controlling the emergence of senescent cells at decidualization.

First, we used an in silico approach to evaluate the kinetic changes of ER stress and UPR-associated genes through the decidualization timecourse. This analysis highlighted *HSPA5*, *ATF6*, *ATF4* and *TXNIP* as abundantly expressed across the process. Moreover, network clustering suggested a specific role for these genes upon decidualization of EnSC during the peri-implantation period. In this sense, *HSPA5* upregulation occurring during decidual differentiation suggests the participation of BiP chaperone to sustain the dramatic increase of protein secretion upon decidualization. Additionally, a predominant expression of *TXNIP* in senescent decidual cells might be associated with the activation of the NLRP3 inflammasome, triggering IL-1β release. In contrast, *ATF6* rise in mature decidual cells subset might account for its key role in the implantation process. In agreement, pharmacological inhibition of ATF6 pathway prevented trophoblast expansion in an in vitro model of implantation^36^. Also, ATF6 levels were found highly expressed in the murine uterus near to the implantation site^37^.

The divergence of EnSC phenotypes into DC and snDC subpopulations was previously characterized during the decidualization process^18,20^. On this basis, we focused on the endoplasmic reticulum chaperone BiP, *HSPA5*, a key regulator of the UPR response. Using an in silico approach, *HSPA5* showed a peak of expression just before the divergence, whereas the knockdown of *HSPA5* in EnSCs impaired the decidualization program. This was evidenced by the downregulation of two canonical differentiation markers, *PRL* and *IGFBP1*. Upon further analysis, we observed that the emergence of both mature and senescent cells was altered. This was confirmed by a lower *IL1RL1* expression, as well as a reduced β-galactosidase activity, which are markers of each subpopulation, respectively. It is worth mentioning that, even though the significant reduction of *HSPA5* mRNA levels occurred just until day 4, downstream effects of silencing were observed mostly at day 8 of the differentiation process. This observation reinforces the idea of *HSPA5* as a biphasic gene, with an early role in the control of the final cell fate of endometrial cells at decidualization.

Adding to the relevance of ER chaperones in the decidualization process, previous reports showed that *HSPA8* might play a role in embryo-quality biosensoring^10^. Moreover, knockdown of *HSPA8* in decidual cells generates an exacerbated UPR, which also compromises the production of *PRL* and *IGFBP1*. Here, the knockdown of *HSPA5* impaired the progression of the decidual pathway and the divergence of cells into DC and snDC subpopulations.

ER stress is triggered upon the accumulation of misfolded proteins inside the endoplasmic reticulum. In the context of decidualization, this is caused by the dramatic increase of secreted factors. Alongside the effect of *HSPA5* knockdown on the decidual markers, we observed the induction of higher levels of ER stress sensor proteins IRE1α and PERK. In the light of these observations, it should be considered whether the impaired decidualization is caused by the absence of BiP itself, or if these effects are due to a stronger UPR response in decidualizing cells. While both scenarios are possible and compatible, it is noteworthy that ER stress levels caused by *HSPA5* knockdown seemed to be moderate, since cell death was not induced.

The role of BiP in cellular senescence is unclear since its levels were found to be increased^38,39^ or decreased^40–44^ depending on the cellular context. Moreover, two non-small cell lung cancer models displayed a different effect on *HSPA5* expression in response to cisplatin induced senescence^45^. Interestingly, in that work Ei et al. showed that cells that upregulated *HSPA5* expression during senescence induction were able to re-enter the cell cycle after cisplatin removal, suggesting a role for BiP in senescence reversal. This observation is particularly interesting in the context of the decidualization process, since the endometrium is a highly dynamic tissue that regenerates itself in each menstrual cycle.

Considering the broad ER stress and UPR pathway, several lines of evidence point to a link with cellular senescence; however, the literature is controversial. A positive association between both processes was reported by Matos et al. using a human diploid fibroblasts model. They observed that ER stress was induced not only by replicative senescence, but also by stress-induced premature senescence. Moreover, senescence induced IRE1α and PERK activation, while the inhibition of these pathways prevented senescence features such as β-galactosidase activity and p21 expression^46^. In line with this, X radiation-induced senescence also triggered ER stress in pulmonary artery endothelial cells^47^. On the contrary, Zhu et al. showed a reduction of ER stress levels under oncogene-induced senescence on mouse keratinocytes; while ER stress induction augmented cell proliferation and attenuated βgalactosidase activity^48^. This opposite association between ER stress and senescence is in line with the results reported here for decidualizing cells, though deeper studies of the molecular mechanisms and specific UPR pathways involved are needed.

From a clinical perspective, alterations in both ER stress and senescence have been associated with alterations in endometrial receptivity and early pregnancy. In this sense, inhibition of IRE1α endonuclease activity in decidualized cells reduced the ability of trophoblast cells to expand over them in an in vitro model^8^. This enzymatic activity controls the cytosolic splicing of XBP1 mRNA, which is a key feature of the UPR process. Interestingly, endometrial samples from patients with Recurrent Implantation Failure show lower levels of the spliced XBP1 mRNA compared to fertile women, suggesting that mild UPR levels are needed for a proper endometrial receptivity^8^. On the other hand, patients with Recurrent Pregnancy Loss (RPL) display an aberrant decidual response as well as uNK cell deficiency, which might be associated to impaired clearance of senescent cells^18^. The accumulation of senescent cells promotes a persistent inflammation via continuous production of the SASP, so-called bystander senescence, which underpins a pathological process with a gradual loss of organ function. In addition, biosensing of soluble signals from low fitness embryos inhibits uNK cell-dependent removal of senescent decidual cells^26^.

The compromised balance between decidualized and senescent cells, as well as altered ER stress levels, might impact endometrial function. In this sense, *HSPA5* knockdown impaired colony-forming unit activity of primary EnSC, indicative of loss of cellular plasticity. Interestingly, it has been shown that RPL is strongly associated with uterine stem cell deficiency, subsequently limiting differentiation potential, disturbing decidualization and leading to consecutive miscarriages^49^. Moreover, decreased decidual senescence in the context of a clinical trial was associated to upregulated clonogenic capacity of EnSCs^50^.

Together, our results show a key role for HSPA5/BiP in reprogramming EnSC and highlight the importance of constraining ER stress levels during this process. Considering the relevance of ER stress and senescence in endometrial homeostasis, as well as their role in fertility complications, the identification of *HSPA5* in the control of the decidual pathway progression sets this chaperone as a potential key regulator of the endometrial function. Further studies are needed to elucidate its role in vivo and its prognostic value for endometrial receptivity.

## Materials and methods

### Endometrial sample collection

Endometrial biopsies were obtained from patients attending the Implantation Clinic at University Hospitals Coventry and Warwickshire (UHCW), Coventry, UK. Samples were obtained during the luteal phase of a non-hormonally stimulated cycle using a Wallach Endocell endometrial sampler. Written informed consent was obtained from patients and all methods were performed in accordance with the Declaration of Helsinki. Collection of endometrial biopsies for research purposes was approved by the NHS National Research Ethics–Hammersmith and Queen Charlotte’s and Chelsea Research Ethics Committee (REC reference: 1997/5065) and Tommy’s National Reproductive Health Biobank (REC reference: 18/WA/0356). Demographic details of the patients are shown in Supplementary Table I.

### Primary EnSC cultures

Endometrial stromal cells (EnSCs) were isolated from endometrial biopsies as described previously^51^. Briefly, samples were mechanically minced with a scalpel for 5 min, followed by an enzymatic digestion with 500 µg/mL of collagenase type Ia (Sigma-Aldrich, UK) and 100 µg/mL of DNase I for 1 h at 37 °C (Lorne Laboratories Ltd, UK). Then, the digested tissue was filtered using a 40 µM cell strainer, and the flow-through was collected and centrifugated at 300 × g. The cell pellet was resuspended in DMEM/F12 (Thermo Scientific, UK) containing 10% dextran-coated charcoal- treated fetal bovine serum (DCC-FBS), 1 × antibiotic-antimycotic mix, 10 µM of L-glutamine (Thermo Scientific, UK), 1 nM of estradiol, and 2 µg/mL of insulin (Sigma-Aldrich, UK) (10% DMEM/F-12). Cells were maintained at 37 °C in a 5% CO_2_ humidified environment and passaged at sub-confluency by treatment with 0.25% Trypsin-EDTA.

Before decidualization experiments, cells were cultured in multi-well plates and media was changed to phenol red free DMEM/F-12 containing 2% DCC, antibiotic/antimycotic and L-glutamine at the concentrations mentioned above (2% DMEM/F12). To induce decidual differentiation, cells were stimulated with 1 µM medroxyprogesterone acetate (MPA) and 0.5 mM 8-bromo-cAMP (SigmaAldrich, UK) for 4 or 8 days as indicated.

### siRNA transfection

EnSCs were plated in 12 or 96-well plates according to the experiment, cultured with 10% DMEM/F-12 media and transfected using jetPRIME Polyplus transfection kit (VWR International, UK) following manufacturer’s instructions. Transfection was performed using 50 nM *HSPA5* siRNA (SigmaAldrich, UK) or 50 nM nontargeting (NT) siRNA as negative control as previously reported^52^. After overnight incubation, transfection mixture was removed, and media was changed to 2% DMEM/F12. Cell viability was immediately tested by XTT assay and bright field microscopy, while decidualization protocol was started 24 h later. Knockdown was confirmed by RTqPCR and Western Blot.

### XTT cell viability assay

Primary EnSC were grown in 96well plates, then transfected and cultured as indicated above. Cell viability was assessed using the XTT cell viability Assay Kit (Biotium Inc.) according to the manufacturer’s instructions. Absorbance values were determined at 0, 1, 2 and 4 h on a PHERAstar FS plate reader (BMG Labtech, Germany) at 450/630 nm. Cells were maintained at 37 °C in a 5% CO_2_ humidified environment in between the measurements. Results were expressed as Fluorescence Intensity Units (F.I.U.).

### Reverse transcription quantitative PCR (RTqPCR)

Primary EnSCs were harvested from 12-well plates and RNA was extracted using RNeasy plus micro kit (Qiagen, UK) according to manufacturer’s instructions. RNA concentration was quantified by Nanodrop spectrophotometer and equal RNA mass was retrotranscribed into cDNA using the QuantiTect Reverse Transcription Kit (Qiagen, UK). Gene expression of the genes of interest was evaluated by RTqPCR on a QuantStudio5 RealTime PCR System (Applied Biosystems, Thermofisher Scientic, Paisley, UK) using QuantiFast SYBR green PCR Mastermix (Qiagen, UK). L19 was used as housekeeping gene and expression level calculated using the ΔCt method. Primer sequences used are shown in Supplementary Table II.

### Senescence associated β-galactosidase activity (SA-β-Gal) quantification

SA-β-Gal was determined using the 96-well Quantitative Cellular Senescence Assay kit (Cell Biolabs Inc., USA). A modified version of the manufacturer’s protocol was used as previously reported^26^. Briefly, EnSC cultured in 96-well plates were washed with ice-cold PBS and lysed in 50 µL of icecold assay lysis buffer containing cOmplete Protease Inhibitor Cocktail (Roche, Switzerland). Cell lysates (25 µL) were transferred to black-walled, black-bottomed 96-well plates, and incubated with 2 × assay buffer. Plates were incubated in a non-humidified, non-CO_2_ incubator for 1 h at 37 °C. Then, stop solution was added and fluorescence was determined on a PHERAstar FS plate reader (BMG Labtech, Germany) at 360/465 nm. Assays were normalized to seeding density and results were expressed as Fluorescence Intensity Units (F.I.U.).

### Western blot analysis

Protein expression of BiP, IRE1a and PERK was evaluated by Western Blot analysis as previously reported^20^. Briefly, EnSCs cultured in 12-well plates were lysed with RIPA buffer containing cOmplete Protease Inhibitor Cocktail (Roche, Switzerland). Cell lysates were harvested and centrifuged (10,000 × *g*, 10 min, 4 °C), supernatants removed, and cell lysates stored at -80 °C until analysis. Protein content was determined by Bradford assay and adjusted to 1 mg/ml with lysis buffer. Samples were heated at 100 °C for 5 min in the presence of NuPage LDS 4 × sample buffer (Fisher Scientific, UK) and 100 nM DTT. Samples were loaded in 8% and 10% mini poly-acrylamide gels and proteins separated by SDS-PAGE electrophoresis. Proteins were then transferred onto 0.45 mm nitrocellulose membranes (GE Healthcare, Amersham, UK). Membranes were blocked with 5% milk in TBS-T buffer (50 mM Tris, 150 mM NaCl, 0.5% Tween- 20, pH 7.4) for 1 h and then probed with primary antibodies targeting BiP, IRE1α, PERK (1:1000; Cell Signaling, USA) and β-actin, (1:100,000; SigmaAldrich, UK). After overnight incubation at 4 °C, blots were washed and incubated with antirabbit-HRP or anti-mouse-HRP conjugated secondary antibody (1:1000 in TBS-T; Agilent) for 1 h at RT. Finally, membranes were washed, and immune-reactive bands were visualized with ECL reagent (GE Healthcare, Amersham, UK). The density of individual bands was determined using Genetools gel analysis software (Syngene, UK).

### In vitro colony-forming unit (CFU) assay

CFU assay was performed as previously reported^49,53^. Briefly, primary EnSC were transfected with either *HSPA5* or NT siRNA overnight and subsequently maintained in either an undifferentiated state or decidualized with C + M for 8 days. Following this, cells were harvested and seeded onto fibronectincoated plates at a density of 1000 cells per well. Cultures were sustained in 10% DMEM/F12 supplemented with 10 ng/ml basic fibroblast growth factor for 10 days, with media replenishment at day 7. Subsequently, wells were rinsed with PBS, fixed in 10% formalin for 10 min at room temperature, and stained with hematoxylin for 5 min. Colonies were visualized using an EVOS AUTO microscope (ThermoFisher Scientific) with a 10 × objective, and images were captured utilizing scan and stitch modalities. Colonies containing at least 50 cells were considered for counting. Cloning efficiency (%) was determined by the ratio of the number of colonies formed to the number of cells seeded, multiplied by 100.

### scRNA-seq in silico analysis

Published scRNA-seq data were obtained from Gene Expression Omnibus (GEO) accession numbers: GSE127918 and GSE247962. Data were integrated and analysed using the standard workflow in Seurat version 4.3.0.1^54,55^ with R version 4.0.3.

### Statistical analysis

Statistical significance of differences between groups was determined using the GraphPad Prism 9 software (GraphPad Software Inc., USA). Wilcoxon rank sum test, One-way ANOVA, Two-way ANOVA or Mixed effect analysis were performed according to the experiment. *P* < 0.05 was considered significant.

## Electronic supplementary material

Below is the link to the electronic supplementary material.

Supplementary Material 1

Supplementary Material 2

Supplementary Material 3

Supplementary Material 4

Supplementary Material 5

Supplementary Material 6

## Data Availability

The datasets used and/or analysed during the current study available from the corresponding author on reasonable request.

## References

[1] Wang, W. et al. Single-cell transcriptomic atlas of the human endometrium during the menstrual cycle. *Nat. Med.* **26**, 1644–1653 (2020). 32929266 10.1038/s41591-020-1040-z

[2] Muter, J., Lynch, V. J., McCoy, R. C. & Brosens, J. J. Human embryo implantation. *Development (Cambridge England)* **150** (2023). 10.1242/dev.201507 PMC10281521 37254877

[3] Dekel, N., Gnainsky, Y., Granot, I. & Mor, G. Inflammation and implantation. *Am. J. Reprod. Immunol.*** 63**, 17–21. 10.1111/j.1600-0897.2009.00792.x (2010). 10.1111/j.1600-0897.2009.00792.x PMC3025807 20059465

[4] Weiss, G., Goldsmith, L. T., Taylor, R. N., Bellet, D. & Taylor, H. S. Inflammation in reproductive disorders. *Reprod. Sci. (Thousand Oaks Calif)*. **16**, 216–229 (2009). 10.1177/1933719108330087 PMC3107847 19208790

[5] Mor, G. & Cardenas, I. The immune system in pregnancy: a unique complexity. *Am. J. Reprod. Immunol.*** 63**, 425–433. 10.1111/j.1600-0897.2010.00836.x (2010). 10.1111/j.1600-0897.2010.00836.x PMC3025805 20367629

[6] Gellersen, B. & Brosens, J. J. Cyclic decidualization of the human endometrium in reproductive health and failure. *Endocr. Rev.* **35**, 851–905 (2014). 25141152 10.1210/er.2014-1045

[7] Walter, P. & Ron, D. The unfolded protein response: from stress pathway to homeostatic regulation. *Science*. 10.1126/science.1209038 (2011). 22116877 10.1126/science.1209038

[8] Grasso, E. et al. Impact of the reticular stress and unfolded protein response on the inflammatory response in endometrial stromal cells. *Sci. Rep.* **8**, 12274 (2018). 30116009 10.1038/s41598-018-29779-8 PMC6095878

[9] Soczewski, E. et al. Immunoregulation of the decidualization program: focus on the endoplasmic reticulum stress. *Reproduction*. 10.1530/REP-19-0391 (2020). 31990665 10.1530/REP-19-0391

[10] Brosens, J. J. et al. Uterine selection of human embryos at Implantation. *Sci. Rep.* **4**, 3894 (2015). 10.1038/srep03894 PMC3915549 24503642

[11] Wang, J., Lee, J., Liem, D. & Ping, P. HSPA5 gene encoding Hsp70 chaperone BiP in the endoplasmic reticulum. *Gene* **618**, 14–23 (2017). 28286085 10.1016/j.gene.2017.03.005 PMC5632570

[12] Grootjans, J., Kaser, A., Kaufman, R. J. & Blumberg, R. S. The unfolded protein response in immunity and inflammation. *Nat. Rev. Immunol.*10.1038/nri.2016.62 (2016). 27346803 10.1038/nri.2016.62 PMC5310224

[13] Guzel, E. et al. Endoplasmic reticulum stress and homeostasis in reproductive physiology and pathology. 10.3390/ijms18040792 (2017). 10.3390/ijms18040792 PMC5412376 28397763

[14] Merksamer, P. I. & Papa, F. R. The UPR and cell fate at a glance. *J. Cell Sci.* **123**, 1003–1006 (2010). 20332117 10.1242/jcs.035832 PMC2844313

[15] Iwawaki, T., Akai, R., Yamanaka, S. & Kohno, K. Function of IRE1 alpha in the placenta is essential for placental development and embryonic viability. *Proc. Natl. Acad. Sci. USA* **106**, 16657–16662 (2009). 19805353 10.1073/pnas.0903775106 PMC2757843

[16] Jiang, Y. H. et al. Serine protease inhibitor 4-(2-aminoethyl)benzenesulfonyl fluoride hydrochloride (AEBSF) inhibits the rat embryo implantation in vivo and interferes with cell adhesion in vitro. *Contraception* **84**, 642–648 (2011). 22078196 10.1016/j.contraception.2011.03.017

[17] Vrljicak, P. et al. Dynamic chromatin remodeling in cycling human endometrium at single-cell level. *Cell Rep.* **42**, 113525 (2023). 38060448 10.1016/j.celrep.2023.113525

[18] Lucas, E. S. et al. Recurrent pregnancy loss is associated with a pro-senescent decidual response during the peri-implantation window. *Commun. Biol.* **3**, 1–14 (2020). 31965050 10.1038/s42003-020-0763-1 PMC6972755

[19] Rawlings, T. M. et al. Modelling the impact of decidual senescence on embryo implantation in human endometrial assembloids. *eLife* **10**, 1–24 (2021). 10.7554/eLife.69603 PMC8523170 34487490

[20] Brighton, P. J. et al. Clearance of senescent decidual cells by uterine natural killer cells in cycling human endometrium. *eLife* **6**, 1–23 (2017). 10.7554/eLife.31274 PMC5724991 29227245

[21] Baar, M. P. et al. Targeted apoptosis of senescent cells restores tissue homeostasis in response to chemotoxicity and aging. *Cell* **169**, 132–147e16 (2017). 28340339 10.1016/j.cell.2017.02.031 PMC5556182

[22] Acosta, J. C. et al. A complex secretory program orchestrated by the inflammasome controls paracrine senescence.* Nat. Cell Biol.* **15**, 978–990 (2014). 10.1038/ncb2784 PMC3732483 23770676

[23] Van Deursen, J. M. The role of senescent cells in ageing. *Nature* **509**, 439–446 (2014). 24848057 10.1038/nature13193 PMC4214092

[24] Deryabin, P., Griukova, A., Nikolsky, N. & Borodkina, A. The link between endometrial stromal cell senescence and decidualization in female fertility: the art of balance. *Cell. Mol. Life Sci.* **77**, 1357–1370 (2020). 31728580 10.1007/s00018-019-03374-0 PMC11104872

[25] Zeng, S. et al. TNFα/TNFR1 signal induces excessive senescence of decidua stromal cells in recurrent pregnancy loss. *J. Reprod. Immunol.* **155**, 103776 (2023). 36495656 10.1016/j.jri.2022.103776

[26] Kong, C. S. et al. Embryo biosensing by uterine natural killer cells determines endometrial fate decisions at implantation. *FASEB J.* **35**, 1–15 (2021). 10.1096/fj.202002217R PMC8251835 33749894

[27] Navid, F. & Colbert, R. A. Causes and consequences of endoplasmic reticulum stress in rheumatic disease. *Nat. Rev. Rheumatol.* **13**, 25–40 (2017). 27904144 10.1038/nrrheum.2016.192

[28] Lerner, A. G. et al. IRE1α induces thioredoxin-interacting protein to activate the NLRP3 inflammasome and promote programmed cell death under irremediable ER stress. *Cell Metab.* **16**, 250–264 (2012). 22883233 10.1016/j.cmet.2012.07.007 PMC4014071

[29] Oslowski, C. M. et al. Thioredoxin-interacting protein mediates ER stress-induced β cell death through initiation of the inflammasome. *Cell Metab.* **16**, 265–273 (2012). 22883234 10.1016/j.cmet.2012.07.005 PMC3418541

[30] Kuroda, K., Ochiai, A. & Brosens, J. J. The actions of resveratrol in decidualizing endometrium: acceleration or inhibition? *Biol. Reprod.* **103**, 1152–1156 (2020). 33029621 10.1093/biolre/ioaa172

[31] Durairaj, R. R. P. et al. Deregulation of the endometrial stromal cell secretome precedes embryo implantation failure. *Mol. Hum. Reprod.* **23**, 478–487 (2017). 28402555 10.1093/molehr/gax023

[32] El-Azzamy, H. et al. Characteristic changes in decidual gene expression signature in spontaneous term parturition. *J. Pathol. Transl. Med.* **51**, 264–283 (2017). 28226203 10.4132/jptm.2016.12.20 PMC5445200

[33] Wijaya, J. C. et al. Functional changes in decidual mesenchymal stem/stromal cells are associated with spontaneous onset of labour. *Mol. Hum. Reprod.* **26**, 636–651 (2021). 10.1093/molehr/gaaa045 32609359

[34] Evans, J. & Salamonsen, L. A. Inflammation, leukocytes and menstruation. *Rev. Endocr. Metab. Disord.*10.1007/s11154-012-9223-7 (2012). 22865231 10.1007/s11154-012-9223-7

[35] Evans, J. & Salamonsen, L. A. Decidualized human endometrial stromal cells are sensors of hormone withdrawal in the menstrual inflammatory cascade. *Biol. Reprod.* **90**, 1–12 (2014). 10.1095/biolreprod.113.108175 24227758

[36] Soczewski, E. et al. VIP conditions human endometrial receptivity by privileging endoplasmic reticulum stress through ATF6α pathway. *Mol. Cell. Endocrinol.* **516** (2020). 10.1016/j.mce.2020.110948 32693008

[37] Xiong, Y. et al. Expression and regulation of ATF6α in the mouse uterus during embryo implantation. *Reprod. Biol. Endocrinol.* **14**, 1–15 (2016). 27717400 10.1186/s12958-016-0199-0 PMC5055674

[38] Liu, J. et al. Receptor for advanced glycation end-products promotes premature senescence of proximal tubular epithelial cells via activation of endoplasmic reticulum stress-dependent p21 signaling. *Cell. Signal.* **26**, 110–121 (2014). 24113348 10.1016/j.cellsig.2013.10.002

[39] Basisty, N. et al. A proteomic atlas of senescence-associated secretomes for aging biomarker development. *SSRN Electron. J.*, 1–26. 10.2139/ssrn.3380253 (2019). 10.1371/journal.pbio.3000599 PMC6964821 31945054

[40] Li, W. et al. Cisplatin-induced senescence in ovarian cancer cells is mediated by GRP78. *Oncol. Rep.* **31**, 2525–2534 (2014). 24756776 10.3892/or.2014.3147

[41] Raghavan, S., Malayaperumal, S., Mohan, V. & Balasubramanyam, M. A comparative study on the cellular stressors in mesenchymal stem cells (MSCs) and pancreatic β-cells under hyperglycemic milieu. *Mol. Cell. Biochem.* **476**, 457–469 (2021). 32997307 10.1007/s11010-020-03922-4

[42] Šrámková, V. et al. Expression of lipogenic markers is decreased in subcutaneous adipose tissue and adipocytes of older women and is negatively linked to GDF15 expression. *J. Physiol. Biochem.* **75**, 253–262 (2019). 30912009 10.1007/s13105-019-00676-6

[43] Wang, T. et al. The ER stress regulator Bip mediates cadmium-induced autophagy and neuronal senescence. *Sci. Rep.* **6**, 1–14 (2016). 27905509 10.1038/srep38091 PMC5131476

[44] Komseli, E. S. et al. A prototypical non-malignant epithelial model to study genome dynamics and concurrently monitor micro-RNAs and proteins in situ during oncogene-induced senescence. *BMC Genom.* **19**, 1–22 (2018). 10.1186/s12864-017-4375-1 PMC5763532 29321003

[45] Ei, Z. Z. et al. GRP78/BiP determines senescence evasion cell fate after cisplatin-based chemotherapy. *Sci. Rep.* **11**, 1–15 (2021). 34789798 10.1038/s41598-021-01540-8 PMC8599848

[46] Matos, L., Gouveia, A. M. & Almeida, H. ER stress response in human cellular models of senescence. *J. Gerontol. Ser. Biol. Sci. Med. Sci.* **70**, 924–935 (2015). 10.1093/gerona/glu129 25149687

[47] Panganiban, R. A. M., Mungunsukh, O. & Day, R. M. X-irradiation induces ER stress, apoptosis, and senescence in pulmonary artery endothelial cells. *Int. J. Radiat. Biol.* **89**, 656–667 (2013). 22788682 10.3109/09553002.2012.711502

[48] Zhu, B. et al. The nuclear receptor peroxisome proliferator-activated receptor-β/ δ (PPARβ/δ) promotes oncogene-induced cellular senescence through repression of endoplasmic reticulum stress. *J. Biol. Chem.* **289**, 20102–20119 (2014). 24898257 10.1074/jbc.M114.551069 PMC4106326

[49] Lucas, E. S. et al. Loss of endometrial plasticity in recurrent pregnancy loss. *Stem Cells* **34**, 346–356 (2016). 26418742 10.1002/stem.2222

[50] Tewary, S. et al. Impact of sitagliptin on endometrial mesenchymal stem-like progenitor cells: A randomised, double-blind placebo-controlled feasibility trial. *EBioMedicine*** 51** (2020). 10.1016/j.ebiom.2019.102597 PMC7000352 31928963

[51] Barros, F., Brosens, J. & Brighton, P. Isolation and primary culture of various cell types from whole human endometrial biopsies. *Bio-Protocol* **6**, 1–13 (2016).27642615

[52] Tissarinen, P. et al. Elevated human placental heat shock protein 5 is associated with spontaneous preterm birth. *Pediatr. Res.* **94**, 520–529 (2023). 36788289 10.1038/s41390-023-02501-9 PMC9926443

[53] Murakami, K. et al. Deficiency in clonogenic endometrial mesenchymal stem cells in obese women with reproductive failure—A pilot study. *PLoS One* **8**, 1–7 (2013). 10.1371/journal.pone.0082582 PMC3858319 24340046

[54] Stuart, T. et al. Comprehensive integration of single-cell data. *Cell* **177**, 1888–1902e21 (2019). 31178118 10.1016/j.cell.2019.05.031 PMC6687398

[55] Hao, Y. et al. Integrated analysis of multimodal single-cell data. *Cell* **184**, 3573–3587e29 (2021). 34062119 10.1016/j.cell.2021.04.048 PMC8238499

