# Supplementary figures and images for: The endoplasmic reticulum protein HSPA5/BiP is essential for decidual transformation of human endometrial stromal cells
Source: Sci Rep. 2024 Oct 29;14:25992. doi: 10.1038/s41598-024-76241-z (PMC11522507; doi:10.1038/s41598-024-76241-z)

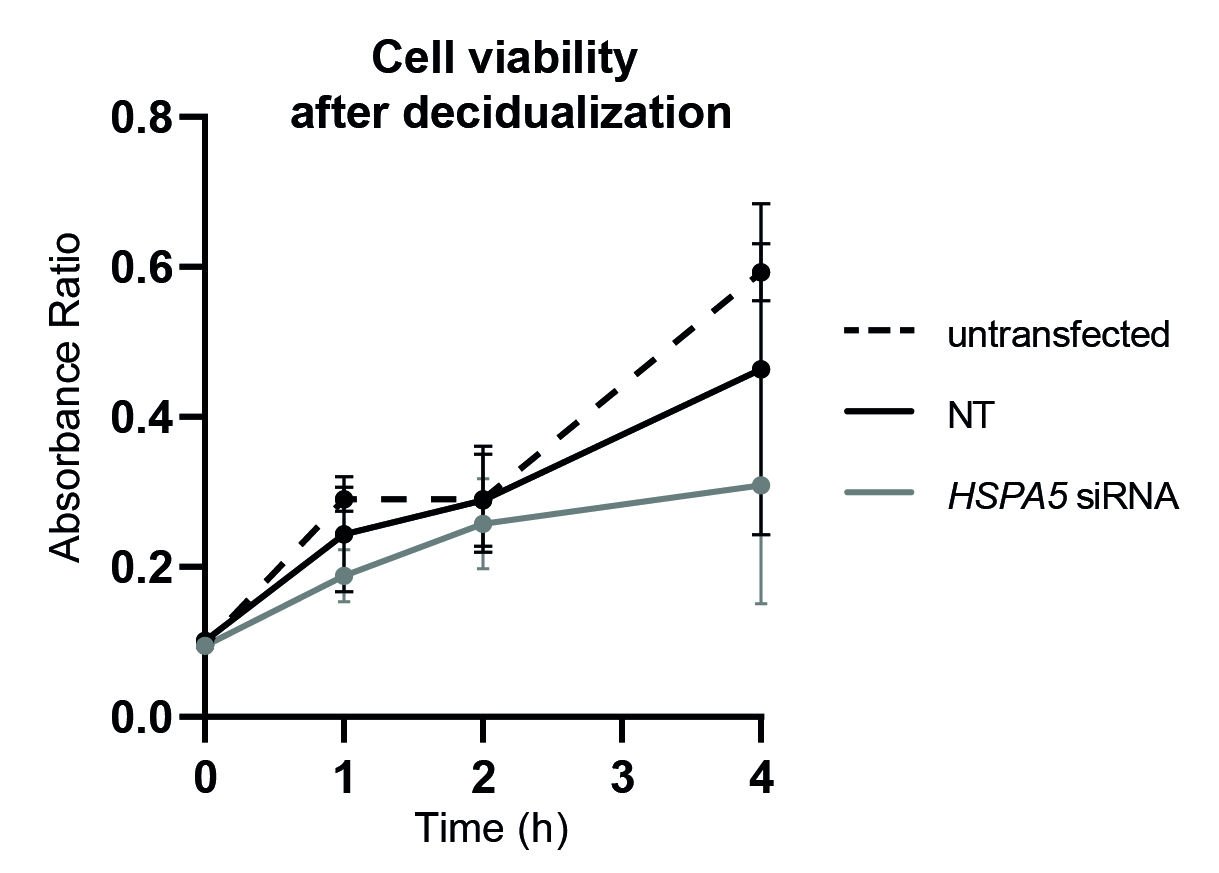

Supplement: Supplementary file 1 — Supplementary Material 1 [file 41598_2024_76241_MOESM1_ESM.jpg]

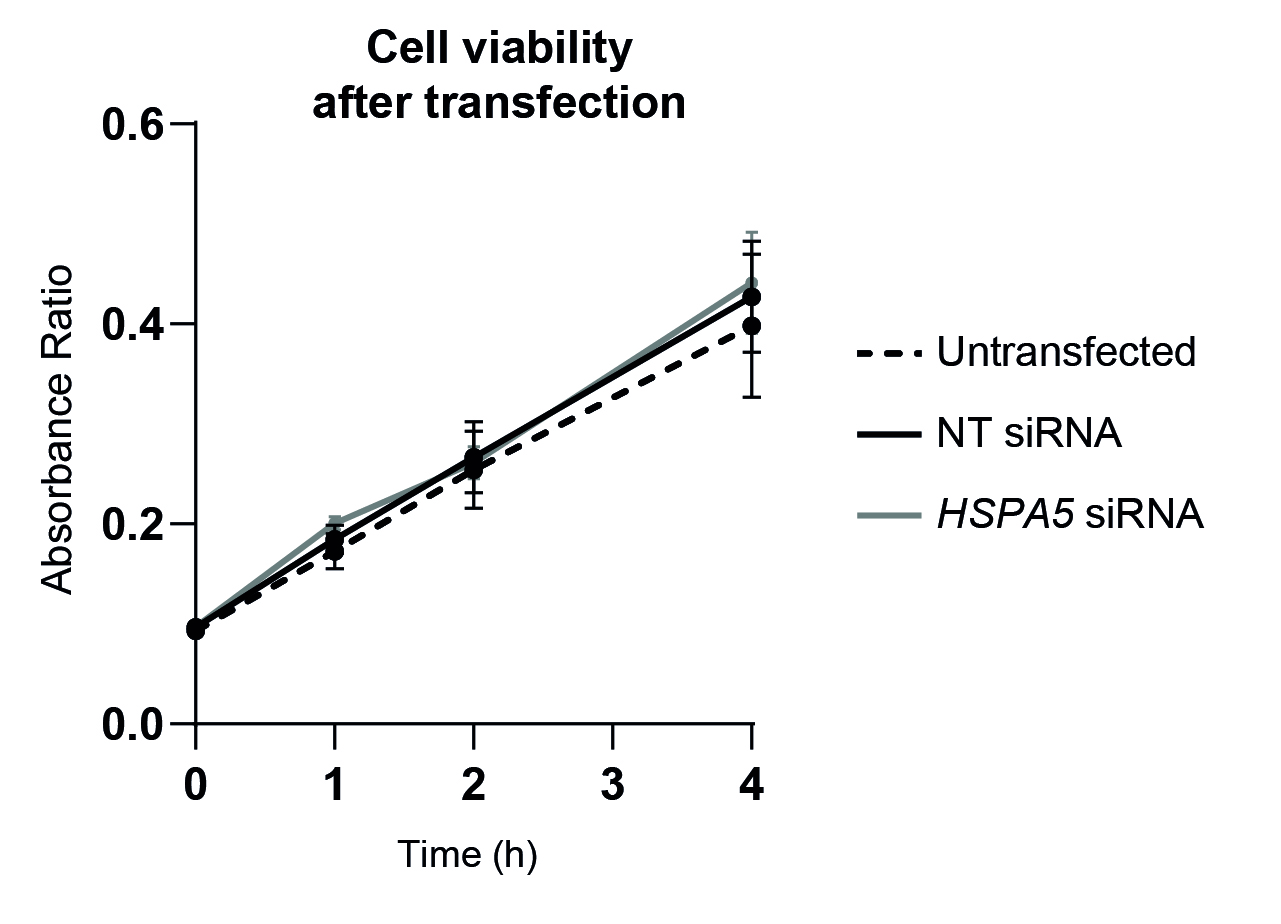

Supplement: Supplementary file 2 — Supplementary Material 2 [file 41598_2024_76241_MOESM2_ESM.jpg]

Original uncropped western blots for figure 3B. Red numbers shown marker band sizes (kDa).

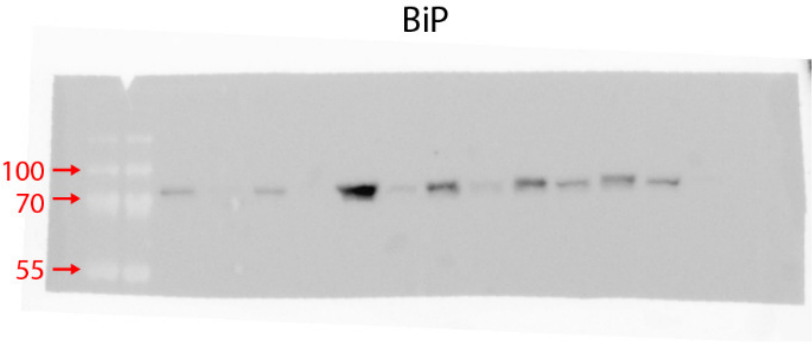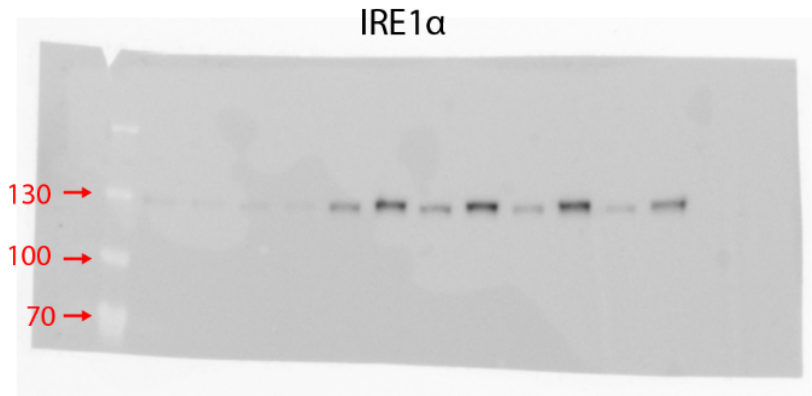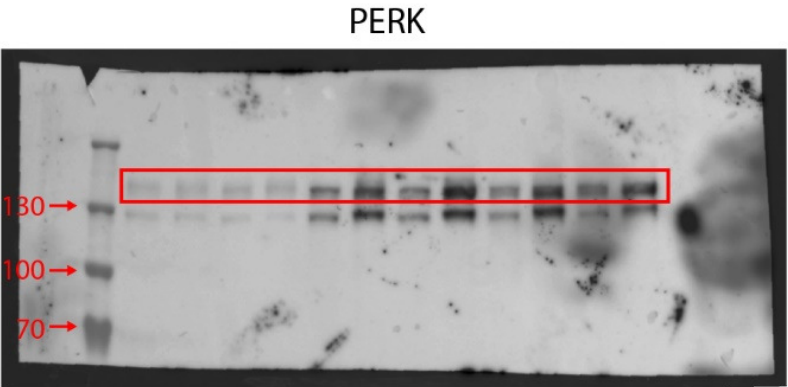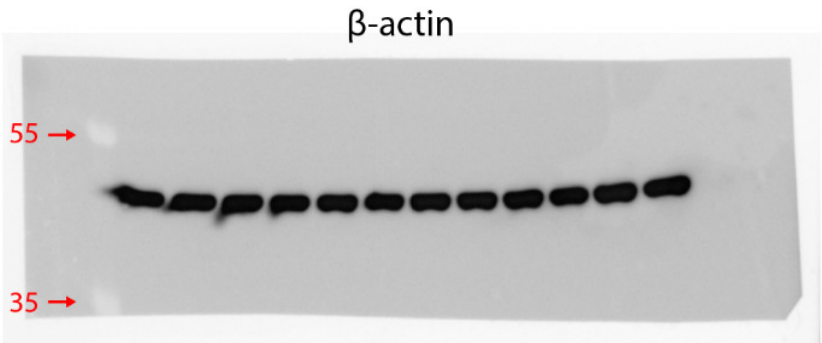

Supplement: Supplementary file 4 — Supplementary Material 4 [file 41598_2024_76241_MOESM4_ESM.pdf]
