# Supplementary material for: The endoplasmic reticulum protein HSPA5/BiP is essential for decidual transformation of human endometrial stromal cells
Source: Sci Rep. 2024 Oct 29;14:25992. doi: 10.1038/s41598-024-76241-z (PMC11522507; doi:10.1038/s41598-024-76241-z)
Supplement: Supplementary file 3 — Supplementary Material 3 [file 41598_2024_76241_MOESM3_ESM.docx]

**Supplementary Figure 1: EnSC viability after siRNA silencing.** EnSCs were transfected with *HSPA5* or NT siRNA. After overnight incubation with the transfection mixture, cell viability was tested by XTT assay. Untransfected cells were used as control. Points and error bars indicate mean±S.E.M. from 4 biological replicates (Two-way ANOVA).

**Supplementary Figure 2: EnSC viability after decidualization.** EnSCs were transfected with *HSPA5* or NT siRNA and then decidualized *in vitro*. Cell viability was tested by XTT assay at decidualization day 8. Untransfected cells were used as control. Points and error bars indicate mean±S.E.M. from 3 biological replicates (Two-way ANOVA).
