# Supplementary material for: The endoplasmic reticulum protein HSPA5/BiP is essential for decidual transformation of human endometrial stromal cells
Source: Sci Rep. 2024 Oct 29;14:25992. doi: 10.1038/s41598-024-76241-z (PMC11522507; doi:10.1038/s41598-024-76241-z)
Supplement: Supplementary file 5 — Supplementary Material 5 [file 41598_2024_76241_MOESM5_ESM.docx]

**Supplementary Table I:** *Patients’ demographic characteristics*.

| **ID** | **Age** | **Live births** | **1st trimester losses** | **BMI** | **Days post LH surge** | **Figures used in** |
| --- | --- | --- | --- | --- | --- | --- |
| 1 | 36 | 0 | 3 | 28 | 7 | 3A / 4D / S1 |
| 2 | 35 | 1 | 5 | 36 | 8 | 3A / 4D / S1 |
| 3 | 37 | 0 | 3 | 20 | 7 | 3A / 4A-D / S1 |
| 4 | 41 | 1 | 3 | 28 | 9 | 3A / 4A-D / S1 |
| 5 | 36 | 0 | 0 | 29 | 7 | 3A,B / 4A-C / S2 |
| 6 | 38 | 0 | 1 | 27 | 7 | 3A,B / 4A-C / S2 |
| 7 | 31 | 0 | 6 | 23 | 8 | 3A / 4A-C / S2 |
| 8 | 37 | 0 | 4 | 25 | 9 | 5A-D |
| 9 | 43 | 0 | 4 | 25 | 8 | 5A-D |
| 10 | 39 | 0 | 0 | 31 | 7 | 5A-D |
| 11 | 30 | 0 | 2 | 23 | 7 | 5A-D |
