# Supplementary material for: The endoplasmic reticulum protein HSPA5/BiP is essential for decidual transformation of human endometrial stromal cells
Source: Sci Rep. 2024 Oct 29;14:25992. doi: 10.1038/s41598-024-76241-z (PMC11522507; doi:10.1038/s41598-024-76241-z)
Supplement: Supplementary file 6 — Supplementary Material 6 [file 41598_2024_76241_MOESM6_ESM.docx]

**Supplementary Table II:** *Primers used in RTqPCR assays.* Oligonucleotide primers were designed using the online tool PrimerBlast (NCBI NIH, USA).

| **Gene** | **Sense (S) or Antisense (AS)** | **Primer sequence (5’ → 3’)** |
| --- | --- | --- |
| *HSPA5* | S | ATC AAC GAG CCT ACG GCA G |
|  | AS | AGA CAC ATC GAA GGT TCC GC |
| *IGFBP1* | S | CGA AGG CTC TCC ATG TCA CCA |
|  | AS | TGT CTC CTG TGC CTT GGC TAA AC |
| *IL1RL1* | S | TTG TCC TAC CAT TGA CCT CTA CAA |
|  | AS | GAT CCT TGA AGA GCC TGA CAA |
| *L19* | S | GCG GAA GGG TAC AGC CAA |
|  | AS | GCA GCC GGC GCA AA |
| *PRL* | S | AAG CTG TAG AGA TTG AGG AGC AAA C |
|  | AS | TCA GGA TGA ACC TGG CTG ACT A |
